# The neural correlates of the visual consciousness in schizophrenia: an fMRI study

**DOI:** 10.1007/s00406-020-01167-2

**Published:** 2020-08-19

**Authors:** S. Lefebvre, E. Very, R. Jardri, M. Horn, A. Yrondi, C. Delmaire, C. Rascle, K. Dujardin, P. Thomas, D. Pins

**Affiliations:** 1 University of Lille, Inserm U1172, Centre Lille Neuroscience and Cognition, CHU Lille, 59000 Lille, France; 2 Plateforme CURE, CHU Lille, Hôpital Fontan, 59000 Lille, France; 3 Translational Research Center, University Hospital of Psychiatry, University of Bern, Bern, Switzerland; 4 Neuroimaging Department, Lille University Medical Center, 59000 Lille, France; 5 Department of Neurology and Movement Disorders, Lille University Medical Center, 59000 Lille, France; 6 ToNIC, Toulouse NeuroImaging Center, Université de Toulouse, INSERM UMR 1214, CHU PURPAN – Pavillon BAUDOT, Place du Dr Joseph Baylac, 31024 Toulouse, France

**Keywords:** Schizophrenia, Consciousness disorder, fMRI, Anterior cingulate cortex, Conscious visual processing, Unconscious visual processing

## Abstract

In the current literature, two distinct and opposite models are suggested to explain the consciousness disorders in schizophrenia. The first one suggests that consciousness disorders rely on a low-level processing deficit, when the second model suggests that consciousness disorders rely on disruption in the ability to consciously access information, with preserved unconscious processing. The current study aims to understand the mechanisms associated with visual consciousness disorder in order to pave the road that will settle the debate regarding these hypotheses. During a functional magnetic resonance imaging session, 19 healthy participants (HC) and 15 patients with schizophrenia (SCZ) performed a visual detection task to compare the neural substrates associated with the conscious access to the visual inputs. The visual detection threshold was significantly higher in SCZ than in HC [*t*(32) = 3.37, *p* = 0.002]. Whole-brain ANOVA demonstrated that around the visual detection threshold patients with SCZ failed to activate a large network of brain areas compared to HC. (1) During conscious vision, HC engaged more the left cuneus and the right occipital cortex than patients with SCZ, (2) during unconscious vision, HC engaged a large network that patients with SCZ failed to activate, and finally, (3) during the access to consciousness process, patients with SCZ failed to activate the anterior cingulate cortex. These results suggest that the consciousness disorders in schizophrenia rely on specific dysfunctions depending on the consciousness stage. The disorders of the conscious vision are associated with dysfunction of occipital areas while the ones associated with unconscious vision rely on a large widespread network. Finally, the conscious access to the visual inputs is impaired by a dysfunction of the anterior cingulate cortex. The current study suggests that none of the two suggested models can explain consciousness disorders in schizophrenia. We suggest that there is an alternative model supporting that the conscious access to visual inputs is due to a disengagement of the supragenual anterior cingulate during the unconscious processing of the visual inputs associated with a sensory deficit.

## Introduction

Visual consciousness is a complex process involving both the processing of visual information by the retina and encoding of that information by a widespread cortical network [1]. However, conscious perception is a graduated process since a visual percept needs to reach a threshold level of perceptual information to attain consciousness [2, 3]. However, even at this threshold, subjective perception can be different. A stimulus with identical physical properties can be either “seen” as a visual stimulus (i.e., reached the threshold of consciousness) or “unseen”. In this second case, the input will remain unconsciously perceived [4, 5]. Crucially, even “unseen”, an unconsciously perceived stimulus involves non-conscious visual processing, as demonstrated by behavioral studies showing that such a stimulus can influence a subject behavior on a subsequent task [6] and by neuroimaging studies demonstrating that visual areas are activated during its presentation [7–10]. By studying this specific period, the visual detection threshold, the phenomenon of access to consciousness of visual inputs can be studied, leading to the exploration of the mechanisms that are associated with the transition from the unconscious process to the conscious one.

In the last 20 years, researchers have tried to identify the neural networks associated with conscious perception using consciously or non-consciously perceived visual stimuli primarily to disentangle the neural activity associated with their respective processing [4]. The actual models agree to consider several levels in consciousness (Temporo-spatial theory of consciousness [11]). The access to consciousness of visual inputs is specifically described in the Global Neuronal Workspace (GNW) [7, 12]. In this model, the visual stimulus information reaches the salience threshold by inducing the activation of the sensory areas (enough bottom-up strength to decode the stimulus) concomitantly to a sufficient involvement of the frontoparietal [4, 8, 13] and insular/cingulate cortices [14] causing a top-down amplification. Thus, a stimulus stays unconscious if the strength of the activation of the sensory areas is not enough, or if the frontoparietal network fails to be activated.

Studying conscious access of visual inputs could be a good way to evaluate consciousness disorders, which in that case, will refer to disturbed processing that leads to intrusive symptoms or abnormal contents into consciousness, meaning that some inputs that would have never become conscious, will reach the consciousness. Surprisingly, the understanding of the neural substrates associated with the access to consciousness of visual inputs is still incomplete in psychiatric diseases, among which schizophrenia (SCZ) seems to be a good model of consciousness disorders. Actually, SCZ is commonly conceptualized as a disorder relying on heterogeneous manifestations that involve fundamental perturbations in consciousness [15–22]. For example, symptoms such as a deficit in memory recollection or awareness of action are associated with consciousness disorders in SCZ [23–25]. Hallucinations, especially present in SCZ and which are, by definition, transient, intrusive and unintentional perceptions in the absence of external sensory stimulation [26–28], are deemed as internal stimuli that would have been not perceived in a large part of the population but reached the consciousness threshold in these patients. So, hallucinations could be due to the conjunction of an impairment of bottom-up processing through sensory cortices [29–32] or a weakening of top-down attentional and monitoring control supported by cingulo-frontal networks [33–37]. Moreover, a theoretical paper also suggested that hallucination disorders are associated with impaired message-passing in the cortical hierarchy [38, 39].

Impairments in consciousness in SCZ as in healthy subjects have been mainly studied in the visual modality. Previous work on consciousness disorders showed, using detection tasks, that the visual detection threshold is higher in patients with SCZ than in healthy participants [40]. Based on neurophysiological data, some authors suggested that this elevated threshold arises from a low-level deficit [41–43]. In contrast, recent studies suggested that the deficit in access to consciousness derived from a disruption in the ability to consciously access and manipulate information with preserved unconscious processing [40, 44]. Interestingly, this deficit seems to rely on a dysfunction of the cingulate cortex especially during conscious processing [45, 46].

Based on the GNW, the aim of the current study is to understand the mechanisms associated with visual consciousness disorder by exploring the conscious access to visual inputs leading to three different stages of processing: (1) conscious visual processing at the visual detection threshold, (2) unconscious visual processing at the visual detection threshold and (3) which neural substrates lead to access to consciousness by exploring the transition between these two processing stages. We aim to pave the road that will settle the debate regarding the two opposite views about the origin of consciousness disorders in schizophrenia. By exploring the conscious access to visual inputs, we will explore if the deficit is relying on (1) a deficit of the unconscious processing (which would involve a dysfunction of the sensory pathway meaning mostly the occipital areas) versus, (2) a deficit of conscious processing (which would involve a dysfunction of the anterior cingulate cortex).

## Materials and methods

### Population

This study received approval from the local investigational review board (CPP Nord-Ouest IV, Lille, France), which followed the provisions of the Declaration of Helsinki. We recruited 15 stabilized patients with SCZ (DSM-IV-R criteria) and 19 healthy participants (HC), all matched for sex and age (Table 1).

**Table 1:** Demographic characteristics of the participants

	Patients with schizophrenia (*n* = 15)	Healthy controls (*n* = 19)	Statistical test	Significance (2 tailed)
Sex: male/female	13/2	17/2	OR = 0.77 (0.04–11.9)	*p* = 1
Age (years)	31.5 (7.4)	31.2 (6.5)	*t*(32) = − 0.13	*p* = 0.89
Handedness: right/left	13/2	16/3	OR = 1.21 (0.11–16.5)	*p* = 1
PANSS				
Positive	20 (6)			
Negative	17 (6)			
General	37 (9)			
Total	75 (16)			
Duration of illness, years	8.7 (5.7)			
CPZ equivalent, mg	416 (474)			

Unless otherwise noted, the scores represent the mean (SD)

All participants were between 18 and 40 years old and had a normal or corrected-to-normal vision. The exclusion criteria were (1) contraindications to MRI, (2) inability to perform the task or to understand the instructions, (3) history of neurological disease or head trauma, (4) history of substance or alcohol misuse, (5) pregnancy, and (6) sensorial or intellectual impairment (IQ < 80). Patients who fulfilled the DSM-IV-R criteria for schizoaffective disorder were not retained. We used the Positive and Negative Syndrome Scale (PANSS; [47]) to assess the psychopathology severity in the patients. All patients were medicated with antipsychotic drugs at the time of testing [see Table 1 for chlorpromazine (CPZ) equivalents].

### Study design

Each participant took part in two separate sessions. In the first session, we assessed the neurological and neuropsychological conditions of all the participants. Then, we trained them on the visual detection task for a brief period. The second session was dedicated to MRI acquisition and was decomposed into three distinct stages: (1) vision correction and selection of MRI-compatible spectacles, (2) familiarization with the MRI environment and the use of an MRI-compatible response pad (Cedrus LU 400-PAIR, Cedrus Corporation, San Pedro, CA 90734, USA), and (3) fMRI data acquisition during the detection task.

### Paradigm

**Fig. 1:**
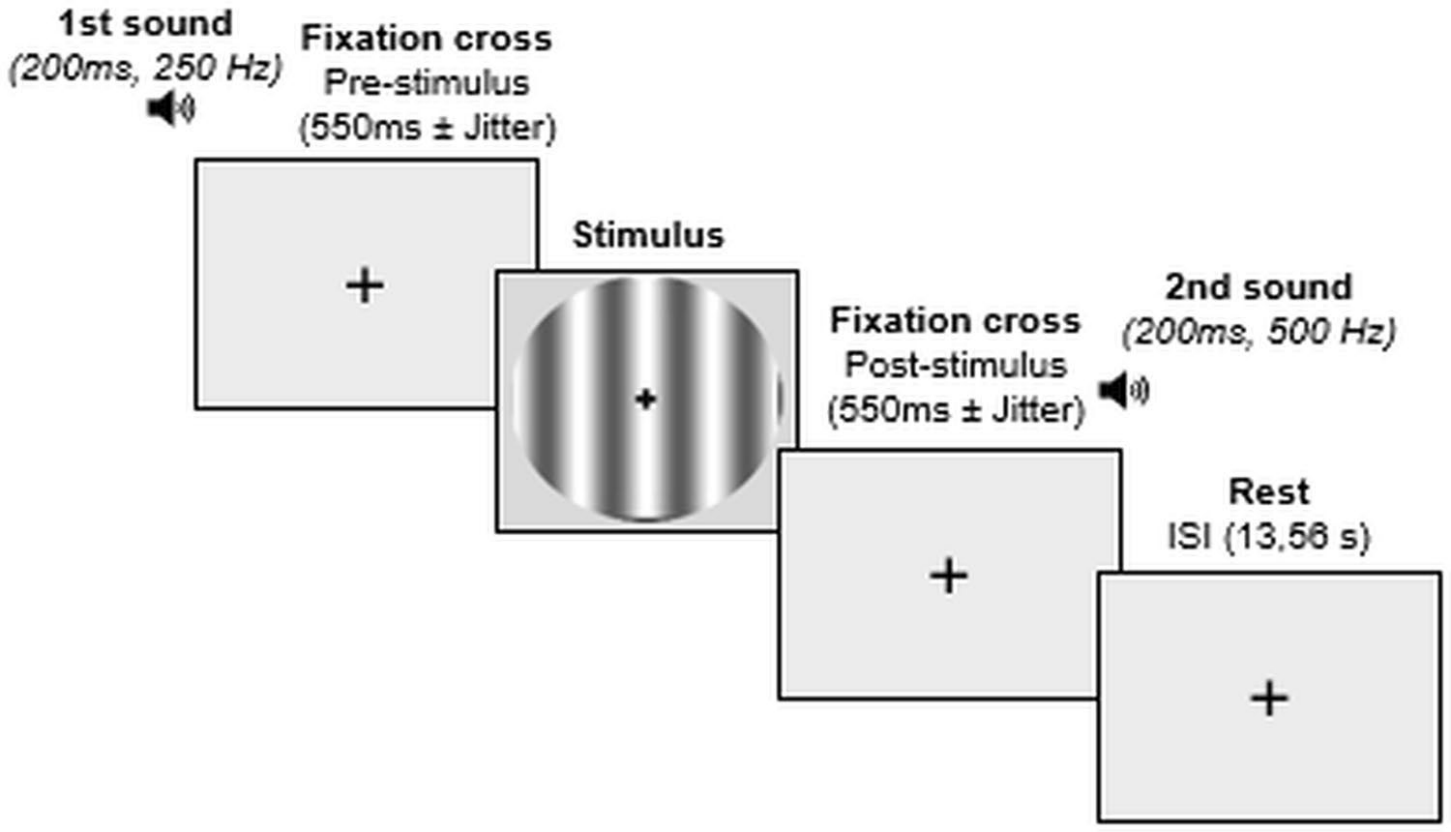
Design. A trial is composed of four steps: (1) a sound (250 Hz, 200 ms) announces the start of the trial; (2) the stimulus appears after an interval of 550 ms plus a random jitter time ranging from 0 to 1100 ms (the pre-stimulus interval), its initial presentation duration is 400 ms, which subsequently decreases or increases over trials based on participant’s answers; (3) a sound (500 Hz, 200 ms) prompts to the participants to provide her/his answer, and (4) an inter-stimulus interval of 13.56 s, using a fixation cross, allows the hemodynamic response to return to baseline between trials

In this study, we specifically evaluated the conscious access to the visual inputs (i.e., the moment when visual stimuli reach consciousness) by using an already validated paradigm in both healthy and neurological populations using a two-alternative forced-choice procedure [48, 49]. Briefly, this procedure relies on 60 trials of a visual detection task that requires the participants to detect a visual stimulus (sinusoidal grating) (Fig. 1).

#### Stimuli

The stimulus was similar to the stimulus used in [48] and consisted of a sinusoidal, circular grating of 1 cycle/degree displayed on a grey background and subtending 10° of visual angle in the center of the visual field. The mean luminance of the grating was 3.5 cd/m^2^, with a contrast of 1.6% (*L*_max_ − *L*_min_/*L*_max_ + *L*_min_). The background luminance was equal to the mean luminance of the grating. A small grey cross in the center of the screen served as a fixation mark to minimize eye movements during the task. During the training sessions, we presented the visual stimulus on a computer monitor (LG Flatron© 17′, 85 Hz refresh rate, a screen-eye distance of 1 m) in a dark room. During the fMRI sessions, we back-projected the visual stimulus onto a translucent screen placed at the end of the scanner bore and was viewed through an angled mirror (LCD projector Toshiba TLP 450E, resolution 1280 × 1024 pixels, refresh rate 75 Hz). We used homemade software (Vision180©) to generate the stimulus.

#### Visual detection task

The task relied on a two-alternative forced-choice procedure to answer the question: “Have you seen the grating?”. We used an adaptative method consisting of changing the duration of the stimulus presentation from trial to trial, based on the participant’s previous response (developed by [48]). The experiment included two descending staircases that were randomly interleaved. Within each staircase, the presentation duration of the grating decreased by ~ 13 ms (equivalent to one screen refresh) for a “Yes” answer and increased by ~ 13 ms for a “No” answer. The stimulus duration changed in steps of 70 ms at the start of each experiment until the first response inversion to allow participants to reach the detection threshold in fewer trials. Using this adaptive method, participants would remain at a 50% detection rate, even if their attention fluctuated during the fMRI session, allowing us to obtain an approximately equal number of trials between seen and unseen gratings (see our previous work with Parkinson’s disease patients for a complete description [49]).

The participants had to press one of the two buttons (“Yes” or “No”) on a response pad with their right hand to indicate whether they had seen the stimulus. The experiment itself contained 60 trials. Among those 60 trials, there were 12 catch trials randomly included throughout the experiment: 6 of them (negative control stimuli) displayed no stimulus (a 0% contrast grating), and 6 (positive control stimuli) displayed an “always seen” grating (100% contrast, 400 ms duration), in order to check for objective errors (false alarms and misses).

Each trial was composed of four steps (Fig. 1): (1) a sound (250 Hz, 200 ms) announced the start of the trial, (2) the stimulus appeared after an interval of 550 ms plus a random jitter time ranging from 0 to 1100 ms (the pre-stimulus interval), its initial presentation duration was set at 400 ms and varied across trials based on participant’s answers, (3) after a post-stimulus interval, a second sound (500 Hz, 200 ms) indicated to the participants to provide her/his answer, and (4) an inter-stimulus interval of 13,56 s, using the fixation cross, which allowed the hemodynamic response to return to baseline between trials. The duration of the grating, itself and the timing of its presentation changed from trial to trial; however, by adjusting the post-stimulus time, the overall trial time remained constant. We displayed the fixation cross for the entire trial, which helped the participants to focus their attention on the middle of the screen.

### Threshold estimation and behavioral analyses

For each participant, we performed a linear regression analysis on each set of 5 consecutive trials, on each staircase respectively. We considered the trials to be at the threshold when the slope of the regression line was zero. We considered all other trials to be “not at the threshold”. Accordingly, there were five categories of trials: (1) gratings seen at the threshold (ST), (2) gratings unseen at the threshold (UT), (3) gratings seen not at the threshold (SNT, including seen positive control stimuli), (4) gratings unseen not at the threshold (UNT), and (5) other trials (OTs, including negative control stimuli and error trials, i.e., when participants failed to reply or made a mistake in positive control trials). We defined the visual detection threshold for each participant as the mean duration of stimulus presentation for trials at the threshold (ST and UT).

To compare visual detection thresholds between HC and patients with SCZ, we used a two-tailed two-sample Student *t* test. We considered a *p* value below 0.05 statistically significant. To assess if the visual detection threshold could be related to the initial PANSS score or the CPZ equivalent doses, we realized an explorative Pearson’s correlation test between these two scores and the visual detection threshold.

### fMRI data acquisition and preprocessing

We acquired a three-dimensional (3D) T1 anatomical scan and a 15-min fMRI scans.

#### Acquisition

We used a 1.5 T MRI scanner (Intera Achieva, Philips Medical Systems, Philips Healthcare P.O., Best, The Netherlands) with an 8-element SENSE head coil to acquire the MRI images including a three-dimensional (3D) T1 anatomical data and fMRI data. The T1-weighted anatomical sequence (3D multi-shot TFE) acquisition consisted of 124 axial slices of 1.6 mm thickness (TR = 8.2 ms, TE = 4 ms, flip angle = 8°, matrix size = 256 × 256, TFE factor = 192, voxel size 1 × 1 mm^2^, reconstructed in 1 × 1 × 1 mm^3^ resolution). The T2*-weighted functional sequence was a single-shot sensitivity-encoded echo-planar imaging sequence (SENSE) with the following parameters: repetition time = 3000 ms, flip angle = 90°, matrix size = 64 × 64, field of view = 240 mm^2^, in-plane resolution = 3.75 × 3.75 mm^2^, slice thickness = 4 mm, number of slices = 38, number of volumes = 304, duration = 15 min.

#### Preprocessing

We used BrainVoyager QX software (Version 2.8, Brain Innovation, Maastricht, The Netherlands) to analyze the MRI data. To remove the non-steady-state effect caused by T1-saturation, we discarded the first four functional volumes, leaving 300 volumes for each participant. The preprocessing of the functional data consisted of a slice timing correction, time-domain high-pass filtering (i.e., removing frequencies below three cycles/run), and 3D motion correction for head movements using a rigid body algorithm. To coregister between functional runs and 3D-T1 weighted scans of each subject we used an automatic gradient-driven affine transformation with nine alignment parameters, and, if needed, we adjusted manually. We applied a spatial normalization into Talairach space [50] and a spatial smoothing using a 6 mm Gaussian filter to all volumes.

### fMRI data analysis

We modeled the functional data using a general linear model (GLM) with predictors based on each of the experimental conditions (ST, UT, SNT, UNT, and OT, in which beta weights measured the predictors’ potential contributions to each voxel time course). We created three distinct GLMs, one for each group and one including all the population sample.

#### Groups RFX (GLM-based)

For each group (HC and SCZ), we reported the functional activation associated with each condition of interest by using the condition estimates (beta values) from a random effect (RFX) GLM analysis for the 15 patients in the SZC groups and the 19 participants for the HC group.

Thus, for each group, we presented (1) conscious vision [ST], (2) unconscious vision [UT], and (3) conscious access to visual inputs [ST–UT], according to the GNW taxonomy [12]. We used a cluster-level corrected threshold using the "ClusterThresh" plugin in BrainVoyager. The computation of the minimum cluster threshold was accomplished via MonteCarlo simulation (1000 repetitions) of the random process of image generation, followed by the injection of spatial correlations between neighboring voxels and voxel intensity thresholding [51, 52]. We adjusted the voxel-level probability threshold, to “*p* < 0.01”, which leads to a minimum cluster size threshold (for each analysis) with an alpha = 5%.

In addition, for the SCZ group, we checked the contribution of medication, age, and PANSS score to the functional activation using medication and PANSS score as covariates.

#### Whole-brain ANOVA (GLM-based)

To directly compare the two groups in terms of the brain activation associated with the presentation of a visual stimulus, we performed a whole-brain, two-factor ANOVA for all the participants (*n* = 34), with trial categories (ST, UT, SNT, UNT, and OT) as the within-group factor and groups as the between-group factor (HC, SCZ). To highlight specific differences, we ran post-hoc analyzes to compare brain activation between the two groups across the three different contrasts of interest (1) conscious vision [ST_HC_ − ST_SCZ_], (2) unconscious vision [UT_HC_ − UT_SCZ_] and (3) conscious access to visual inputs [ST_SCZ_–UT_SCZ_]—[ST_HC_–UT_HC_]. We used a cluster-level corrected threshold with the minimum cluster threshold estimated using MonteCarlo simulations.

#### Region of interest (ROI) analysis

To explore the potential differences in brain recruitment during conscious access to visual inputs between the two groups that might not be revealed by the two-way ANOVA, we performed an ROI analysis. Based on the GLM-RFX, we extracted the beta weights of the ROIs activated for the entire sample during the conscious access to visual inputs [ST–UT] (corrected at the cluster level with the minimum cluster threshold estimated using MonteCarlo simulations*)* and we conducted external comparisons of these beta weights between the two groups using two-tailed two-sample Student *t* tests.

## Results

### Behavior

**Fig. 2:**
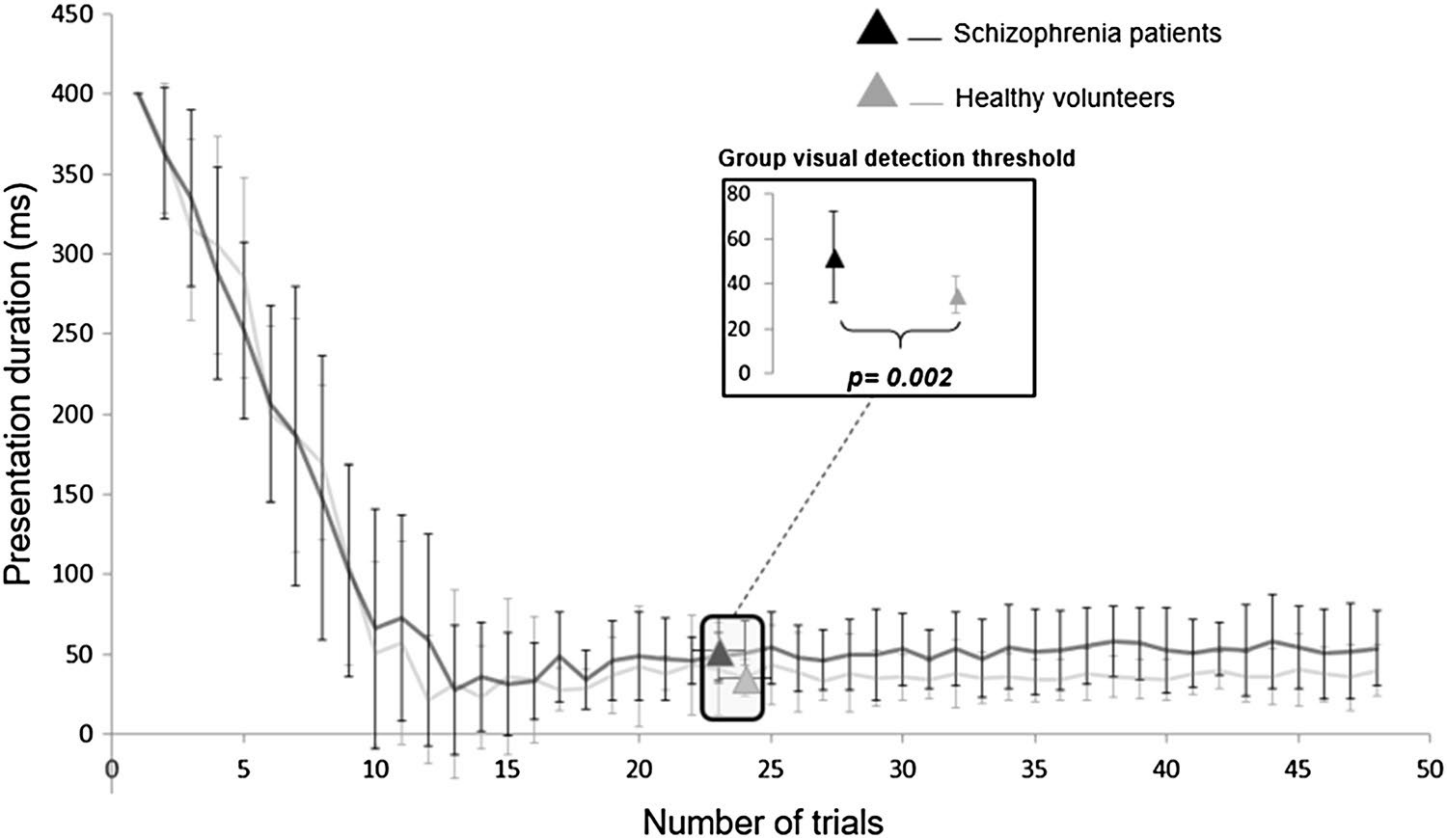
The visual detection threshold. Mean ± SD grating presentation duration (ms) for each of the 48 trials and for both groups (black for schizophrenia patients and grey for healthy volunteers). At trial 23/24, the actual visual detection threshold for each group can be observed. In addition, the comparison of the visual detection threshold between the two groups can be seen in the small add-on “group visual detection threshold”

The visual detection threshold (see Fig. 2) was significantly lower in the HC group (35.08 ± 8.16 ms) than in the SCZ group (52.17 ± 20.20 ms) [*t(*32) = 3.37, *p* = 0.002]. Moreover, the threshold estimation experiment produced an approximately equal number of “seen” and “unseen” trials at the threshold for the 2 groups, with respectively 14.1 ± 1.6 ST and 15.5 ± 2 UT trials on average for the HC group and 13.9 ± 2 ST trials and 15.2 ± 2.7 UT trials on average for the SCZ group. An analysis of catch trials showed that misses (positive control stimuli not seen) and false alarms (negative control stimuli detected) were negligible in both groups. In the HC group, there was an average of 0.16 ± 0.69 misses (out of 6 trials) and 0.32 ± 0.82 false alarms (out of 6 trials) whereas, in the SCZ group, there was an average of 0.07 ± 0.26 misses and 0.07 ± 0.26 false alarms. Moreover, there was no significant correlation between either the initial PANSS score (*r* = 0.21, *p* = 0.47) or the CPZ equivalent doses (*r* = − 0.09, *p* = 0.75) and the visual detection threshold in the SCZ group.

### fMRI results

#### Group RFX

The correlation analyses demonstrated no significant association between any of the covariates (the PANSS score, the age, the medication) and the brain activity associated with the visual detection task in the SCZ group.

**Fig. 3:**
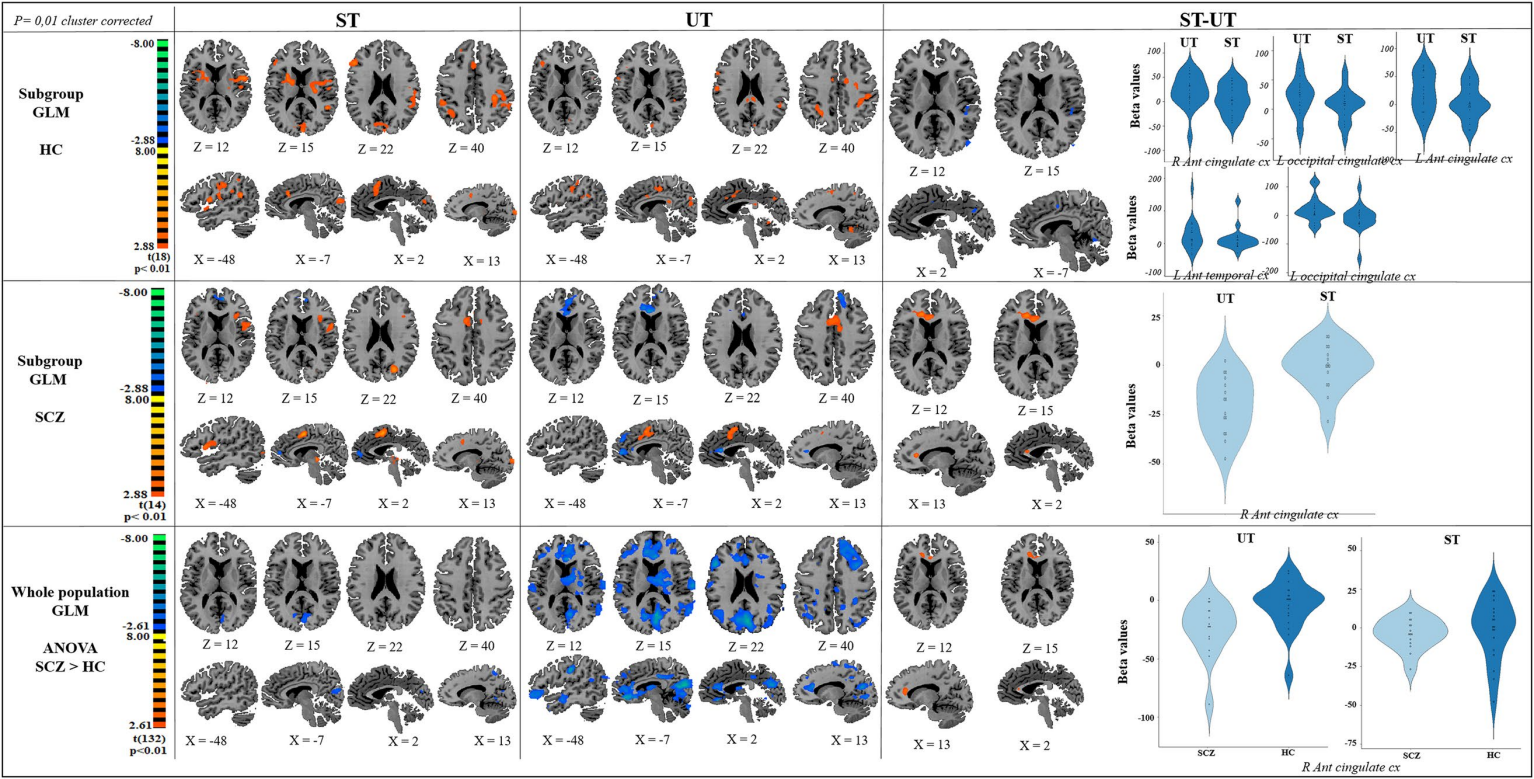
Brain maps for each group and ANOVA comparison. In this display, we report the brain maps for the [ST], [UT] and [ST–UT] contrasts for the HC and SCZ groups and the comparison. For the conscious access to visual input [ST–UT] contrast, we also report violin distributions (light blue for SCZ, dark blue for HC) for the conscious [ST] and unconscious [UT] visual processing

The brain activation observed in each group for each of the three contrasts of interest ([ST] reflecting the conscious visual processing, (UT) reflecting the unconscious visual processing, and [ST–UT] reflecting the neural substrates associated with the conscious access to visual inputs are presented in Fig. 3 and Table 3).

During the conscious visual processing, the HC group activated a large widespread network encompassing bilateral occipital cortices, bilateral insula, bilateral parietal cortices, right prefrontal cortex, left fusiform cortex, and left somatosensory cortex. The SCZ group activated bilateral occipital cortices, bilateral insula, right superior temporal cortex, and right supplementary motor area (SMA), and deactivated the right prefrontal cortex.

During the unconscious visual processing, the HC group activated bilateral parietal cortices, bilateral SMA, left occipital cortex, left premotor cortex, left cerebellum, and left somatosensory. The SCZ group activated the SMA bilaterally and deactivated the left prefrontal cortex and right anterior cingulate cortex.

The conscious access to visual inputs the HC group relied on small clusters into bilateral anterior cingulate cortex, bilateral occipital cortex and left anterior temporal cortex. These clusters were more involved during the unconscious visual processing than conscious visual processing. In the SCZ group, the conscious access to visual inputs relied on the anterior cingulate cortex which tends to be deactivated during the unconscious visual processing.

#### Whole-brain ANOVA

##### Main effects

Whole-brain ANOVA revealed significant main effects of the within-group factor [trial categories (ST, UT, SNT, UNT, OT)], the between-group factor [HC, SCZ] and of the interaction between the within and between-group factors [*p* = 0.01 cluster size corrected, within-factor (trials categories) *F*(4,128) = 3.47; between-factor (groups) *F*(1, 32) = 7.50; interaction (trials categories × groups) *F*(4,128) = 3,47].

##### Post-hoc analyzes

*Comparison of the conscious processing between the SCZ and HC groups* When comparing the neural substrates associated with conscious vision between the HC and SCZ groups ([ST_HC_-ST_SCZ_]) [*t*(132) = 2.61, *p* = 0.01 corrected at the cluster-level, with a minimum cluster size of 675 voxels], we observed a differential activation in two brain areas: the left cuneus and the right occipital cortex (Fig. 3, Table 2). These areas exhibited a strong activation in the HC group whereas patients with SCZ failed to activate them as much as HC during conscious vision. No area showed more activation in the SCZ group than in the HC group ([ST_SCZ_ − ST_HC_]).

**Table 2:** Brain activation associated with each condition for each group

		Brain area	BA	Mean *X*	Mean *Y*	Mean *Z*	mm^3^	*T*	*p*
Conscious vision at the visual detection threshold	HC [ST] (min cluster size 1350 voxels)	R DLPFC	9	47	30	20	1411	4.75	< 0.001
		R parietal cx	39	36	− 52	42	2555	4.17	< 0.001
		R DLPFC	9	31	36	34	1521	4.07	< 0.001
		L insula	13	40	3	10	1920	4.84	< 0.001
		R occipital cx	19	19	− 58	− 6	1399	4.94	< 0.001
		Bil occipital cx	18	0	− 79	19	2105	4.12	< 0.001
		L fusifom cx	37	− 28	− 55	− 9	1353	3.68	0.002
		R insula	13	− 40	− 3	10	3568	4.79	< 0.001
		L S1 cx	3	− 34	− 33	45	4774	5.21	< 0.001
		L parietal cx	40	− 51	− 30	21	3322	5.16	< 0.001
	SCZ [ST] (min cluster size 1215 voxels)	R sup temporal cx	22	53	− 27	2	1490	4.82	< 0.001
		R insula	13	34	17	3	2552	3.16	0.005
		R occipital cx	18	22	− 92	2	2809	1.92	0.06
		R SMA cx	6	1	3	49	6683	4.10	< 0.001
		R prefrontal cx	10	0	51	7	1321	− 3.06	0.007
		L occipital cx	18	− 28	− 84	− 1	4458	4.52	< 0.001
		L occipital cx	19	− 22	− 71	24	1477	2.28	0.04
		L insula	13	− 38	12	6	6 026	4.80	< 0.001
	[ST _SCZ_ − ST_HC]_ (min cluster size 675 voxels)	R occipital cx	19	19	− 58	0	912	− 3.54	< 0.001
		L cuneus	18	− 3	− 74	17	1394	− 3.72	< 0.001
	[ST_SCZ_ − ST _HC]_	Ø							
Unconscious vision at the visual detection threshold	HC [UT] (min cluster size 600 voxels)	R parietal cx	7	26	− 51	47	2744	4.75	< 0.001
		R SMA	6	9	− 6	52	1418	3.67	< 0.001
		L cerebellum		− 25	− 52	− 20	720	4.42	< 0.001
		L occipital cx	19	− 22	− 62	− 7	1180	4.42	< 0.001
		L SMA	6	− 8	− 6	47	1994	4.30	< 0.001
		L premotor cx	6	− 27	− 15	54	1517	5.15	< 0.001
		L parietal cx	7	− 25	− 60	50	660	4.08	< 0.001
		L S1	3	− 32	− 36	48	5389	4.48	< 0.001
	SCZ [UT] (min cluster size 1215 voxels)	R ant cingular cx	32/24	1	38	12	3120	− 5.69	< 0.001
		Bil SMA	6	0	3	47	6788	6.20	< 0.001
		Left prefrontal cx	8	− 13	36	40	3550	− 5.73	< 0.001
	[UT_SCZ_ − UT _HC]_ (min cluster size 675 voxels)	R PMd	6	51	− 11	31	1785	− 4.59	< 0.001
		R prefrontal cx	9	33	12	31	1315	− 4.48	< 0.001
		R parietal cx	7	22	− 44	57	2976	− 4.39	< 0.001
		R inf parietal cx	40	39	− 56	38	942	− 5.49	< 0.001
		R temporal lobe	39	43	− 61	21	1507	− 6.78	< 0.001
		L post cingular cx	31	− 5	− 35	36	2504	− 5.13	< 0.001
		L frontal cx	8	− 20	31	39	7564	− 6.49	< 0.001
		L parietal cx	7	− 24	− 46	52	2606	− 5.22	< 0.001
		L temporal lobe	39	− 41	− 64	21	1731	− 5.05	< 0.001
		L temporal lobe	22	− 56	− 57	15	849	− 4.42	< 0.001
		Bil ant cingulate cx	32/24	− 5	24	10	30,907	− 6.46	< 0.001
		Bil occipital cx	18	− 8	− 71	0	20,306	− 4.51	< 0.001
	[UT_SCZ_ − UT_HC]_	Ø							
Access to consciousness	HC [ST–UT] (min cluster size 100 voxels)	R ant cingulate cx	24	7	− 2	43	115	− 3.45	0.003
		L occipital cx	18	− 1	− 68	29	285	− 3.64	0.002
		L ant cingulate cx	24	− 10	− 4	43	273	− 4.40	< 0.001
		L ant temporal cx	41	− 45	− 32	12	241	− 3.56	0.002
		L occipital cx	19	− 47	− 77	12	192	− 3.45	0.003
	SCZ [ST–UT] (min cluster size voxels)	R ant cingulate cx	32	9	29	16	1090	4.86	< 0.001
	[(ST_SCZ_–UT_SCZ_) − (ST_HC_–UT_HC_)] (min cluster size 675 voxels)	R ant cingulate cx	32	12	32	17	1223	4.15	< 0.001
	[(ST_HC_–UT_HC_) − (ST_scz_–UT_scz_)]	Ø							

*HC* healthy controls, *SCZ* schizophrenia, *BA* Brodmann area, *R* right, *L* left, *PMd* dorsal premotor cortex, *cx* cortex, *inf* inferior, *post* posterior, *ant* anterior, *Bil* bilateral

*Comparison of the unconscious processing between SCZ and HC groups* When comparing the neural substrates associated with unconscious vision between the HC and SCZ groups ([UT_HC_–UT_SCZ_]) [*t*(132) = 2.61, *p* = 0.01 corrected at the cluster level, with a minimum cluster size of 675 voxels], we observed a differential activation in a large widespread network, encompassing the premotor area, prefrontal cortex, and parietal cortex in the right hemisphere; the posterior cingulate cortex, frontal cortex, parietal cortex and temporal lobe in the left hemisphere; the bilateral supragenual anterior cingulate cortex (sgACC) and the occipital cortex. These areas were less involved in the SCZ group compared to HC during the unconscious visual processing (Fig. 3, Table 2). No area showed more activation in the SCZ group than in the HC group ([UT_SCZ–UTHC_]).

*Comparison of conscious access to visual inputs [ST–UT] between the SCZ and HC groups* When comparing the neural substrates associated with the conscious access to visual inputs between SCZ and HC groups [*t*(132) = 2.61, *p* = 0.01 corrected at the cluster-level, with a minimum cluster size of 675 voxels] (Fig. 3, Table 2), we observed a differential activation of the right sgACC. The exploration of the beta values for each condition permitted us to distinguish the effect of each processing in this contrast. In the conscious access to visual inputs, the two groups seemed to only differ for the unseen trials at the threshold [HC: 5.17 ± 18.3, SCZ: − 12.65 ± 11.79, *t*(32) = 3.27, *p* = 0.003]. This suggests that the difference between the two groups in the conscious access to visual inputs resulted from a disengagement of the sgACC in the SCZ group during the unconscious visual processing.

#### ROI analysis

**Fig. 4:**
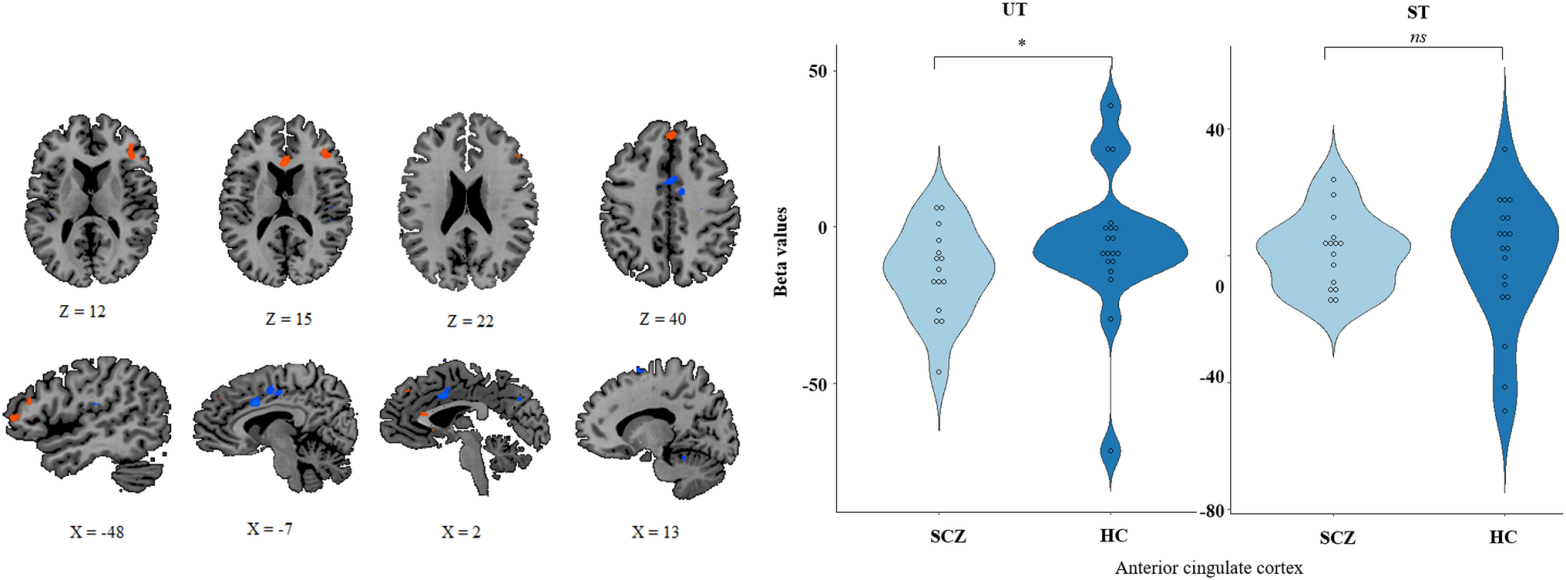
Entire group ST–UT. In this display, we report the brain map for the whole population during conscious access to visual input [ST–UT]. For the only area presenting a group difference (anterior cingulate cortex, see Table 3), we also report violin distributions (light blue for SCZ, dark blue for HC) for the conscious [ST] and unconscious [UT] visual processing. [*t*(32) = 2.73, *p* < 0.01 corrected at the cluster level, with a minimum cluster size of 200 voxels]

We used the global GLM (including the whole population HC + SCZ), and focused on the constrast exploring the conscious access to visual inputs ([ST–UT]). In the whole population, the conscious access to visual inputs relied on a large network of brain areas [*t*(32) = 2.73, *p* = 0.01, corrected at the cluster level, with a minimum cluster size of 200 voxels] encompassing the right sgACC, left DLPFC, supplementary motor area and left parietal cortex (Fig. 4, Table 3a). The comparison of the extracted beta weights of each of these ROIs, confirmed that the unique source of the significant difference between the HC and the SCZ groups during the conscious access to visual inputs was the sgACC (*x* = 1, *y* = 27, *z* = 17, mm^3^ = 209) [*t*(32) = 2.237, *p* = 0.03]. (See Table 3b for group comparison for each ROI). In this specific ROI, there were no significant difference in the beta values between the two groups for the ST trials [HC: 0.94 ± 20.2, SCZ: − 1.51 ± 10.3, *t*(32) = 0.42, *p* = 0.67], whereas, there was a significant difference between the two groups for the UT trials [HC: − 2.9 ± 20.9, SCZ: − 23.5, *t*(32) = 2.68, *p* = 0.0, with a minimum cluster size of 200 voxels] (see Fig. 4). This additional analysis confirmed the previous findings that the group difference in the conscious access to visual inputs resulted from a disengagement of the sgACC in the SCZ group during the unconscious visual processing.

**Table 3:** Whole population access to consciousness of visual inputs (ST–UT)

(a)	Brain area	BA	Mean *X*	Mean *Y*	Mean *Z*	mm^3^	*t*	*p*
ST–UT	R inf parietal cortex	40	34	− 41	47	203	− 3.89	< 0.001
	SMA	6	1	6	42	1930	− 4.34	< 0.001
	Anterior cingulate cortex	32	1	27	17	209	3.45	0.002
	R prefrontal cortex	8	− 1	42	42	270	3.20	0.003
	L PMd	6	− 16	20	50	227	3.47	0.001
	L M1	4	− 37	− 29	53	421	− 3.46	0.001
	L DLPFC	46	− 45	33	11	1132	3.75	< 0.001

(b)	Student *t* test comparison between groups for [ST–UT]			
	Beta values HC (mean)	Beta values SCZ (mean)	*t*	*p*
R inf parietal cortex	− 10.41	− 9.36	0.15	0.89
SMA	− 13.41	− 14.38	− 0.13	0.89
Anterior cingulate cortex	3.85	21.97	2.24	0.03
R prefrontal cortex	24.91	25.32	0.03	0.98
L PMd	8.91	14.03	0.91	0.37
L M1	− 17.34	− 11.94	0.56	0.55
L DLPFC	12.86	16.78	0.60	0.55

Here we report the brain activation associated with the access to consciousness of visual input the entire group (HC + SCZ, *n* = 34). [t(32) = 2.73, *p* < 0.01 corrected at the cluster level, with a minimum cluster size of 200 voxels]

A—Size and statistics for each cluster

B—In each of the ROIs, we realized a Student *t* test to test for group difference. The anterior cingulate cortex, is only area presenting a significant difference between group

*HC* healthy controls, *SCZ* schizophrenia, *BA* Brodmann area, *R* right, *L* left, *PMd* dorsal premotor cortex, *inf* inferior, *SMA* supplementary motor area, *M1* primary motor area, *DLPFC* dorsolateral prefrontal cortex, *mm*^*3*^ number of activated voxels, which corresponds also to the volume in cubic (Talairach) millimeter. Mean *X*, *Y*, *Z* correspond to the center of gravity of the volume of interest. *t* and *p* refer to the statistics of the cluster

## Discussion

The present study showed that patients with SCZ had a higher visual detection threshold than HCs that seems to rely on a reorganization of the brain network associated with conscious access to visual inputs. In particular, SCZ patients presented an altered activation pattern of the sgACC that mostly occurred during the unconscious processing of visual information.

Currently, as already pointed out, two opposing theories have been suggested to explain consciousness disorders in SCZ. The first model suggests that the elevated visual detection threshold in SCZ is linked to dysfunction of the visual processing [41–43], when the second model suggests an impaired top-down amplification [40, 45, 46, 53, 54] which relies on a dysfunction of the cingulate cortex during conscious visual processing [45, 46]. This second model also suggests that patients with SCZ would be able to normally process unconscious stimuli while they would fail to process stimuli that had reached consciousness in healthy controls [44]. These stimuli would not cross the threshold for conscious perception and would remain preconscious/unconscious in SCZ patients. The results presented in the current study suggest an alternative model supporting that the low-level deficit is concomitant to a dysfunction of the sgACC, especially during the unconscious visual processing.

In our study, patients showed functional abnormalities of visual processing at the visual detection threshold when compared to age-matched healthy subjects in both conscious and unconscious conditions. Indeed, during conscious vision, patients with SCZ, compared to healthy subjects, strongly failed to activate the extrastriate cortex, including the cuneus and the visual cortex. Visual processing deficits are well documented in SCZ (for review, see [55]). In particular, numerous studies have reported reduced fMRI BOLD responses in visual areas during various vision paradigms [56–59]. Moreover, patients with SCZ failed to activate the lateral occipital complex for both seen and unseen stimuli during a visual backward masking task [60]. This impaired visual processing in SCZ has also been described in behavioral paradigm, in which patients presented disruption of unconscious semantic priming [61], in electrophysiological data, which demonstrated deficits in early-stages of visual processing [38], or using SCZ post-mortem data, which observed a reduced number of neurons as well as a reduction of the primary visual cortex volume [62]. The present study not only confirms previous evidence that patients with SCZ failed to engage sensory areas during conscious processing of a visual stimulus but also provides further evidence that this deficit can occur during unconscious vision processing as well, supporting the broader hypothesis that deficits in cognitive processing could be driven by impairments in basic perceptual processes that take place to primary sensory brain regions [63].

In addition, as hypothesized by Dehaene, et al. [45], our results also suggest that the impaired access to consciousness in SCZ seems to rely on a dysfunction of the ACC region. In addition to its well-known involvement in a wide range of cognitive functions such as working memory [64], cognitive control [65], conflict and error monitoring [66], top-down attentional processes [67, 68], and emotion processing [69], the ACC also seems to play a key role in perceptual consciousness. Several studies have suggested this less commonly described function. First, anatomical data show that the ACC receives visual inputs from the thalamus and provides reciprocal connections with extrastriate, parietal and prefrontal cortices [7, 70]. Those long-range cortico-cortical connections are likely to provide anatomical roots suitable for a consciousness neural network [7]. Second, robust evidence shows that the ACC is a crucial node within the fronto-parietal network associated with conscious access to visual stimuli [10, 14, 71, 72]. Finally, several authors have proposed that the ACC could have a strong top-down influence on sensory processes, by modulating the stimulus selection for access to consciousness [7, 10]. In the present study, patients suffering from SCZ presented an altered functional pattern of the sgACC. This area is mainly involved in conflict and error monitoring [66, 73, 74], such as when deciding whether a stimulus has been displayed or not, and in top–down attentional processes [67, 68].

Thus, our results provide some clues that when compared to healthy subjects, SCZ patients seemed to fail to engage the large widespread cortical network including the visual areas and the sgACC that is involved in healthy population during unconscious visual processing. These results tend to contradict the hypothesis of a preserved unconscious visual processing in SCZ [44]. Consequently, our combined results suggest a global disorder that includes both low- and high-level deficits. The impoverished ascending processing of visual stimuli, which includes a lower recruitment of the occipital areas, is not enough to efficiently activate the global workspace (which includes fronto-parietal areas and the sgACC) [75], leading to the lack of sgACC activation observed during the conscious access to visual inputs. Thus, these two associated deficits prevent the stimulus from crossing the consciousness threshold leading to impairment of the descending and amplifying attentional processes. Therefore, visual stimuli, which are perceived in healthy subjects, are maintained in an unconscious state in the patients with SCZ resulting in higher visual detection thresholds. Finally, when sensory areas are sufficiently activated, they trigger the global workspace’s activation, leading the visual stimuli to pass the threshold. This model needs to be confirmed by future effective connectivity studies.

The present results tend to differ from the outcomes of previous studies working visual conscious access in SCZ. One of the reasons could be due to the task used. First, our detection task, unlike the one used by instance by Berkovitch, Del Cul et al. [53], assumes that the participants mobilized their attention for each trial. Indeed, the stimulus was always expected and in each trial needed to focus the attentional load between the two sounds which indicated that the stimulus could arrive (first sound) or that it will not come anymore (second sound). Second, unlike our visual detection task, most of the tasks previously used to study a possible conscious/unconscious deficit in patients with SCZ, required participants to manipulate or mainly categorize the perceived stimuli (e.g., say if the stimulus is greater or less than 5…). For example, Dehaene, Artigues et al. [45] found a large hypo-activation of the cingulate cortex during conscious visual processing only when patients performed a complex motor conflict task. Our detection task has the advantage of not requiring any manipulation or additional cognitive processing of the stimuli. This could explain why, when stimuli are consciously perceived, only sensory regions differentiated between healthy subjects and patients.

Accordingly, consciousness disorders seem to be associated with the dysfunction of the cingulate cortex in the conscious or unconscious process, depending on the task performed or the cognitive load. However, another explanation could be that consciousness disorders relied on the dysconnectivity phenomena in SCZ [76]. In SCZ, a dysconnectivity of large-scale networks explains the occurrence of hallucinations, which we already defined as a good representation of consciousness disorders by bringing to consciousness stimuli that would have never reached the consciousness threshold [76]. In a recent paper, we demonstrated that the occurrence of hallucinations relied on an initial dysfunction of the salience network [77]. As the cingulate cortex is part of this complex network, the lack of activation of the cingulate cortex during unconscious visual processing could be related to the global dysfunction of the salience network in SCZ.

The present study had several limitations. Firstly, the acquisitions were performed using a 1.5 T MRI scanner and a relatively long repetition time (TR = 3 s) for an event-related design. Secondly, the enrolled schizophrenia population was quite heterogeneous and the sample size modest. This may have contributed to reduced statistical power and the lack of correlations between imaging and clinical data. However, this paradigm has already been validated in both healthy and neurological populations using similar sample size and similar thresholds [48, 49] and despite this small sample size, we were able to highlight neural activation differences between healthy and SCZ patients. Moreover, a detailed evaluation of the hallucination disorder is lacking in the current sample. The study would benefit to be rerun using more up-to-date imaging parameters (e.g. 3 T, 64 head-coil channel and TR between 1 and 2 s), and a larger population with different clinical subtypes of patients (including a range from absence to severe hallucinations). Thirdly, one could argue that patients showed worse performance and lower brain activity because they were less engaged within the task, rather than indicating a pathological mechanism of consciousness. Indeed, our design did not include a continuous performance monitoring, but the patients did not make more mistakes than the healthy controls on the control trials. Thus, it seems quite unlikely that they were at the same timeless engaged but still correct in answering. However, in future studies, we recommend testing the level of engagement of the patient in the task and maybe having more control trials.

## Conclusion

The present study suggests that consciousness disorders in schizophrenia include both low- and high-level deficits as they relied on both dysfunctions of the sensory pathway and the anterior cingulate. Moreover, our results suggest a strong impairment of unconscious visual processing during access to consciousness. These results open new considerations on understanding consciousness disorders in schizophrenia.
